# Enhancement of Male Sterility Stability in *Indica* Rice by Dual Thermo-Sensitive Genic Male Sterile Genes

**DOI:** 10.3390/plants15121906

**Published:** 2026-06-19

**Authors:** Mingji Wu, Chonghui Ji, Bo Ling, Shaohua Yang, Jianglong Yang, Danli Sun, Menger Zhong, Feng Wang, Wenli Zou, Yiwang Zhu

**Affiliations:** 1 Fujian Provincial Key Laboratory of Agricultural Genetic Engineering, Biotechnology Research Institute, Fujian Academy of Agricultural Sciences, Fuzhou 350003, China; wmj@fjage.org (M.W.); 15375822208@163.com (B.L.); ysh@fjage.org (S.Y.); yjl2722482878@163.com (J.Y.); sundl0409@163.com (D.S.); wf@fjage.org (F.W.); 2 Center for Genomics and Biotechnology, Fujian Provincial Key Laboratory of Haixia Plant Systems Biology, Haixia Institute of Science and Technology, College of Life Sciences, Fujian Agriculture and Forestry University, Fuzhou 350002, China; jichonghui@fafu.edu.cn (C.J.); mongerzhong@163.com (M.Z.); 3 School of Life Sciences, Jiangxi Science & Technology Normal University, Nanchang 330013, China

**Keywords:** CRISPR/Cas9, thermo-sensitive genic male sterility, *tms5*, *MS1*, fertility stability, *indica*

## Abstract

Low-temperature-induced fertility restoration in thermo-sensitive genic male sterile (TGMS) lines severely impairs hybrid seed purity, which is a major bottleneck for two-line hybrid rice production. Most commercial TGMS lines rely on the single *tms5* locus, leading to high climatic vulnerability. In this study, we developed a dual-locus strategy by target genome editing of *TMS5* and *MS1* in *indica* rice GH89. Adenine base editing at the *MS1* locus exhibited a high editing efficiency of 93.5%. Transgene-free homozygous single mutants (GH89-*tms5* and GH89-*MS1*) and double mutant (GH89-*tms5* + *MS1)* were generated for phenotypic analysis. The double mutant GH89-*tms5* + *MS1* remained completely sterile for 5 and 10 days under controlled low temperature (23.5 °C), with only minimal fertility restoration after 15 days. In the field, it maintained complete sterility for 84 consecutive days and was fully insensitive to short-term low temperature fluctuations, outperforming single mutants and commercial control Y58S. Moreover, the double mutant retained most key yield-related agronomic traits of the wild type with only minor variations. This dual mutation forms a “double-lock” fertility regulatory system, significantly increasing the low-temperature duration threshold for fertility restoration. The GH89-*tms5* + *MS1* line exhibits promising potential for future rice breeding applications.

## 1. Introduction

Thermo-sensitive genic male sterile (TGMS) lines are the cornerstone of two-line hybrid rice systems, offering advantages such as flexible parental combination and accelerated breeding cycles [1,2,3]. However, a critical limitation in commercial seed production is the vulnerability of these lines to low-temperature stress. Episodes of low temperature during the fertility-sensitive period can induce partial fertility restoration and self-pollinated seed setting, leading to severe reductions in hybrid seed purity and potential production failure [4,5]. This issue has become a major constraint on the sustainable development of the hybrid rice industry, particularly in the context of global climate change and increased frequency of extreme weather events.

Currently, over 95% of commercially used *TGMS* lines rely on the single genetic locus *tms5* [6,7,8]. *TMS5* encodes RNase Z^S1^, which regulates the degradation of Ub_L40_ mRNA during the pollen mother cell meiosis stage [9]. While effective, the narrow genetic base centered on *tms5* results in a uniform fertility-sensitive window, making the entire two-line system susceptible to synchronized low-temperature risks. To address this limitation, researchers have identified alternative *TGMS* loci, such as *OsMS1*, which encodes a histone-binding protein that regulates tapetum development and pollen formation after meiosis [10]. A natural thermosensitive allele of *OsMS1*, designated *OsMS1*^wenmin1^, confers temperature-sensitive male sterility by altering protein localization and stability in a temperature-dependent manner [11]. Importantly, the fertility-sensitive period of *OsMS1*^wenmin1^ is completely non-overlapping with that of *tms5*, providing a theoretical basis for improving sterility stability through gene pyramiding.

In this study, we hypothesized that stacking *tms5* and the temperature-sensitive allele of *OsMS1*^wenmin1^ (hereafter referred to as *MS1*) would create a “double-lock” regulatory mechanism for fertility control. To precisely introduce these dual mutations into the elite *indica* rice variety GH89, we employed adenine base editing (ABE) and CRISPR/Cas9 technologies, which have been extensively optimized for plant genome editing [12,13]. By combining CRISPR/Cas9 and adenine base editing (ABE) technologies, we introduced these dual mutations into the elite *indica* rice variety GH89, and systematically compared the fertility performance of single and double mutants under both natural field conditions and controlled environments. Our results demonstrate that the dual mutant exhibits remarkable tolerance to low-temperature-induced fertility restoration, providing a robust strategy to enhance the stability and safety of two-line hybrid rice seed production.

## 2. Results

### 2.1. Generation of tms5 and MS1 Single and Double Mutants

**Figure 1:**
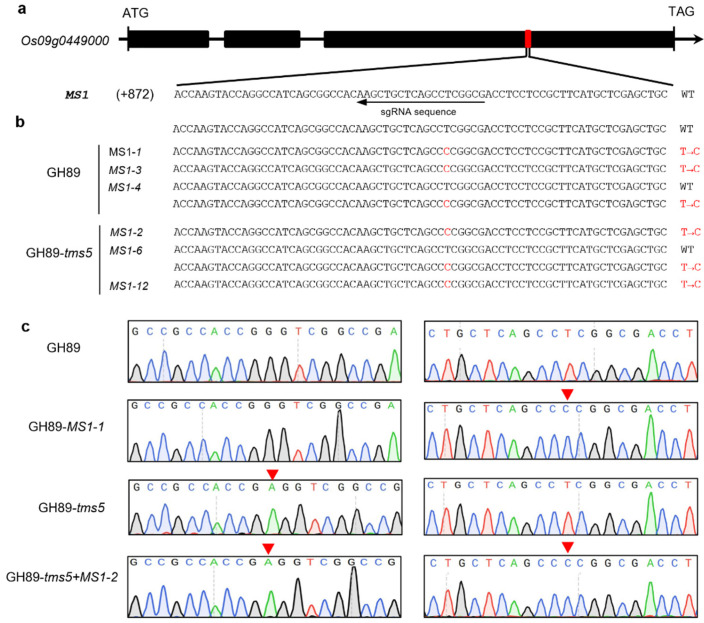
Generation and molecular identification of *tms5* and *MS1* single and double mutants in the *indica* rice variety GH89. (**a**) Schematic diagram of the target site for adenine base editor (ABE)-mediated T → C editing of *MS1* (*Os09g0449000*) in the GH89 genome. Exons of *MS1* are represented by black boxes, and the single-guide RNA (sgRNA) target sequence is indicated by the black arrow. (**b**) Alignment of partial target mutated genomic sequences of ABE-edited MS1 constructs (GH89-*MS1* and GH89-*tms5* + *MS1*) and corresponding wild-type GH89 and GH89-*tms5* sequences. The T → C nucleotide substitutions are highlighted in red, and the wild-type sequence is indicated as “WT”. (**c**) Sanger sequencing chromatograms confirming the transgene-free homozygous T → C base conversion at the *MS1* target site in GH89-*MS1* and GH89-*tms5* + *MS1*, compared with the wild-type *MS1* allele in GH89 and GH89-*tms5*. The red triangles indicate the positions of the T → C substitution in the edited alleles.

*Agrobacterium*-mediated transformation yielded 31 T_0_ positive seedlings, including 14 derived from GH89 and 17 from GH89-*tms5* (Table 1). Sanger sequencing confirmed that all positive plants carried the desired T → C single-base substitution at the *MS1* target site, consistent with the *OsMS1*^wenmin1^ allele. The overall editing efficiency was 93.5%, with 21 heterozygous (T/C) and 8 homozygous (C/C) mutants identified in the T_0_ generation. After screening the T_1_ generation for segregation of the *hygromycin phosphotransferase* (*HPT*) selection marker, we successfully obtained independent homozygous and transgene-free GH89-*MS1* single mutants (GH89-*MS1-1* and GH89-*MS1-3*) and GH89-*tms5* + *MS1* double mutants (GH89-*tms5* + *MS1-2* and GH89-*tms5* + *MS1-12*) for subsequent phenotypic characterization (Figure 1).

### 2.2. Temperature-Dependent Fertility Restoration in tms5, MS1 Single, and Double Mutants Under Controlled Conditions

**Figure 2:**
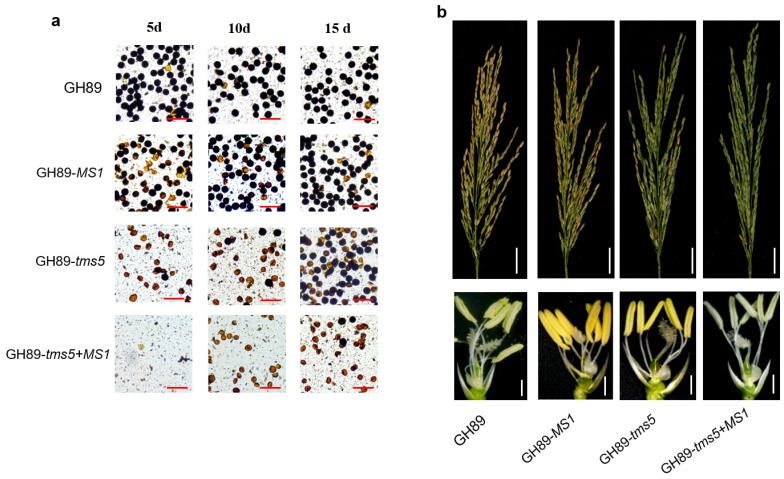
Phenotypic characterization of fertility transition in mutants after low-temperature treatment in an artificial climate chamber. (**a**) Pollen staining (I_2_-KI) of WT, GH89-*tms5*, GH89-*MS1*, and GH89-*tms5* + *MS1* after 5, 10, and 15 days of low-temperature treatment. Scale bars: 80 μm. (**b**) Spike fertility and anther morphology of WT, GH89-*MS1*, GH89-*tms5*, and GH89-*tms5* + *MS1* after 10 days of treatment at 23.5 °C. The double mutant shows pale white, shrunken anthers and sterile panicles, whereas single mutants and WT show partially fertile anthers and panicles. Scale bars: 5 mm [**upper**] and Scale bars: 1 mm [**lower**].

To characterize the temperature-sensitive fertility restoration of the *tms5*, *MS1* single mutants and *tms5* + *MS1* double-gene mutants, temperature-controlled treatments were performed in an artificial climate chamber with an average daily temperature of 23.5 °C. Treatments lasted 5, 10, and 15 days during the young panicle differentiation stage, which corresponds to the thermo-sensitive phase for fertility alteration. Pollen viability and self-pollinated seed setting rate were assessed as indicators of fertility restoration. The results demonstrated that the fertility restoration dynamics clearly distinguished the different genotypes under the controlled low-temperature treatment (Table 2). The *MS1* single mutant GH89-*MS1* was the most sensitive, exhibiting a pollen viability rate of 41.8 ± 16.6% to 45.3 ± 13.8% and a seed-setting rate of 23.6 ± 7.8% to 28.5 ± 15.3% after only 5 days of treatment. The *tms5* single mutants (GH89-*tms5-1;3*) showed moderate tolerance, producing a small amount of fertile pollen but exhibiting no self-pollinated seed setting after 5 days of treatment, while partial fertility restoration was observed after 10 day (Figure 2a; Table 2).

Notably, the *tms5 MS1* double mutants displayed the strongest tolerance to low temperatures. They remained completely male sterile after 5 and 10 days of continuous low-temperature treatment, with both the pollen viability rate and seed-setting rate recorded at 0% (Figure 2a; Table 2). Even after an extended 15-day treatment, fertility restoration was minimal, with a pollen fertility rate of only 10.1 ± 2.5% to 11.8 ± 5.6% and a self-pollinated seed setting rate of 2.1 ± 1.5% to 2.5 ± 1.7% (Figure 2a; Table 2). Consistent with the phenotypic observations, 10 days of low-temperature treatment in the artificial climate chamber revealed distinct differences in spike fertility, anther morphology, and pollen staining among the genotypes (Figure 2b). The double mutant GH89-*tms5* + *MS1* exhibited pale white, shrunken anthers, and the vast majority of its pollen grains were unstained and aborted, confirming complete sterility. In contrast, the single mutants GH89-*tms5* and GH89-*MS1* showed partially fertile anthers and a significant proportion of iodine-stained pollen grains, indicating partial fertility restoration under the same conditions. These results confirm that the dual mutation significantly elevates the low-temperature duration threshold required for fertility restoration.

### 2.3. Male Sterility Stability of tms5, MS1 Single, and Double Mutants Under Natural Field Conditions

Field observations revealed significant genotypic differences in male sterility stability among GH89 mutant lines and the control line Y58S under natural conditions (Table 3). During the early fertile period (6–18 June), all lines exhibited varying degrees of fertility. The GH89-*tms5* mutant showed similar fertility to Y58S, with pollen viability exceeding 60% and self-pollinated seed setting rates ranging from 21.9 ± 10.6% to 51.8 ± 28.9%, meeting the requirements for sterile line propagation. From 24 June to 15 September, both GH89-*tms5* and Y58S remained largely sterile, except for brief fertility recoveries on July 24 and 27, with low pollen viability (6.6 ± 3.3–9.8 ± 6.4%) and minimal seed set (0.5 ± 0.2–1.8 ± 0.9%). In contrast, the GH89-*MS1* single mutant displayed unstable fertility throughout the observation period, indicating sensitivity to environmental fluctuations. Notably, the GH89-*tms5 + MS1* double mutant exhibited the most stable sterility. From 6 to 21 June, it showed partial fertility, but pollen viability (1.3 ± 3.6–38.2 ± 15.6%) was significantly lower than that of the single mutants and Y58S. By June 24, it became completely sterile and remained so for 84 consecutive days until 16 September, with no detectable fertile pollen or seed set at any time point.

Meteorological data (Table 3 and Table S1) indicated that temperature was the key factor influencing fertility transition. Before 6 June, average daily temperatures below 24 °C with the minimum nighttime temperature lower than 22 °C promoted fertility in all lines. From mid-June onward, rising and fluctuating temperatures led to differential responses: GH89-*MS1* was highly sensitive to brief low-temperature intervals, recovering fertility rapidly; GH89-*tms5* and Y58S required more stringent low-temperature conditions, specifically an average daily temperature below 24 °C sustained for three consecutive days, to achieve partial fertility recovery. In contrast, the *tms5* + *MS1* double mutant remained completely sterile throughout the entire period, even during short-term low-temperature events in July, demonstrating superior sterility stability and higher biosafety for two-line hybrid rice breeding.

Correlation and regression verified significant negative temperature-pollen fertility correlations: GH89-*tms5* (*r* = −0.807, *p* < 0.001, *R*^2^ = 0.651), GH89-*MS1* (*r* = −0.675, *p* < 0.001, *R*^2^ = 0.455), GH89-*tms5* + *MS1* (*r* = −0.863, *p* < 0.001, *R*^2^ = 0.745), with the double mutant showing the most robust temperature response.

### 2.4. Agronomic Performance of the Double Mutant Under Natural Field Conditions

**Figure 3:**
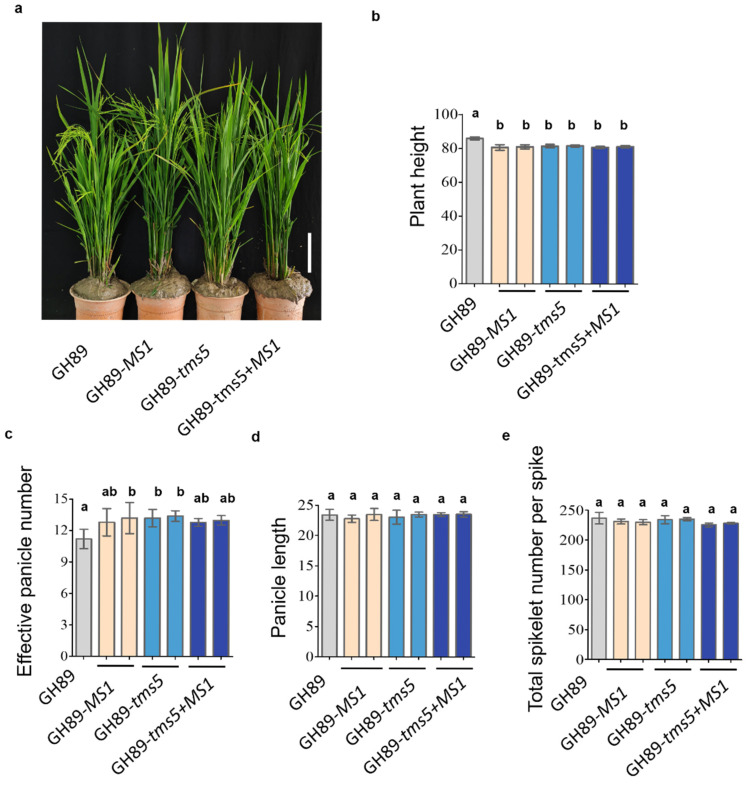
Agronomic performance of wild-type GH89 and mutant lines under natural field conditions. (**a**) Plant morphology of wild-type GH89, GH89-*MS1*, GH89-*tms5*, and GH89-*tms5* + *MS1* under natural field conditions. Scale bars: 20 cm. (**b**–**e**) Quantitative comparison of key agronomic traits, including plant height (**b**), effective panicle number (**c**), panicle length (**d**), and total spikelet number per panicle (**e**), among wild-type GH89 and mutant lines. Two independent homozygous lines were examined for every single mutant and double mutant genotype to ensure phenotypic repeatability. Data are presented as the mean ± SD (*n* = 5). One-way ANOVA followed by Tukey’s multiple comparison test was used to analyze the differences among groups. Different lowercase letters indicate significant differences at *p* < 0.05, while the same letter indicates no significant difference. No significant negative effects on yield-related traits were observed in the mutant lines compared with the wild type.

To assess potential impacts of the mutations on yield-related traits, we further investigated and compared a suite of agronomic traits among the homozygous single mutants GH89-*tms5*, GH89-*MS1*, the double mutant GH89-*tms5* + *MS1*, and wild-type plants under natural field conditions. The key traits analyzed included plant height, effective panicle number per plant, main panicle length, and total spikelet number per panicle (Figure 3). Field observations confirmed that, aside from the difference in pollen fertility, the analysis of these core yield-related traits revealed no significant negative effects in the mutant lines compared to the wild type: plant height was slightly reduced in the mutants, while effective panicle number, main panicle length, and total spikelet number per panicle were maintained at levels comparable to or slightly higher than those of the wild type (Figure 3). These results indicate that the mutations conferring thermo-sensitive male sterility do not impair the fundamental growth and yield component traits of the rice plants, suggesting that the double mutant could serve as a valuable male sterile line in hybrid rice breeding without compromising agronomic performance.

## 3. Discussion

The study presents a novel genetic improvement strategy for *indica* TGMS lines based on the pyramiding of *tms5* and a temperature-sensitive *MS1* allele, and the resulting dual mutant exhibits exceptional male sterility stability and strong tolerance to low temperatures (Figure 2 and Figure 3). The innovation of this strategy lies in the rational selection of two temperature-sensitive sterile loci with non-overlapping fertility-sensitive periods, which is hypothesized to potentially avoids the functional redundancy of stacked loci and maximizes the synergistic effect of fertility regulation. Unlike previous studies that focused on modifying the expression level of a single sterile gene or identifying natural mutant alleles, our approach establishes a synthetic dual-locus regulatory system through precise genome editing, achieving a more robust and controllable fertility control effect. The results show that this system not only raises the threshold of low-temperature duration required for fertility restoration under controlled artificial conditions but also renders the TGMS line entirely insensitive to short-term low-temperature stress in the field environments. These findings provide a new genetic basis for improving the stability and reliability of two-line hybrid rice seed production.

The GH89-*tms5* + *MS1* dual mutant developed in this study holds great practical significance. The mutant has both excellent sterility stability and agronomic compatibility (Figure 2 and Figure 3), making it a directly usable germplasm resource for two-line *indica* hybrid rice breeding. The high editing efficiency of 93.5% achieved in this study also validates the applicability and feasibility of combining CRISPR/Cas9 and adenine base editing for the development of multi-gene mutant TGMS lines.

Although this study has achieved significant progress in enhancing the male sterility stability of *indica* TGMS lines, several aspects warrant further investigation. First, the fertility performance of the GH89*-tms5* + *MS1* dual mutant should be evaluated in different ecological regions with distinct temperature regimes to clarify its adaptability and planting range. Second, it is feasible to explore stacking more temperature-sensitive sterile loci with non-overlapping sensitive periods into the dual mutant to further improve its low-temperature tolerance. In addition, the application of this gene pyramiding strategy to japonica TGMS lines is of great value—*japonica* rice also faces the problem of low-temperature-induced fertility restoration in seed production, and the extended application of this strategy is expected to boost the development of two-line *indica*-*japonica* hybrids. Overall, this study provides an efficient and scalable molecular breeding strategy for improving the male sterility stability of TGMS lines, and the germplasm resource generated here shows strong potential for application in hybrid rice breeding programs.

## 4. Materials and Methods

### 4.1. Plant Materials

The *indica* rice variety GH89 (Guanghui 89) is an excellent intermediate breeding line selected from the progeny of Zhongguang 8A/Minhui 3301. It possesses excellent agronomic traits suitable for being transformed into two-line male sterile lines, including strong tillering ability, favorable flowering habit, high stigma exsertion rate, slender grain shape, superior rice quality, moderate growth period and high yield potential. Consequently, GH89 was used as the wild-type (WT) recipient material. This material is a breeding intermediate line independently bred and long-term preserved in our laboratory, which possesses the characteristics of an elite indica restorer line with high combining ability, strong disease resistance and excellent grain quality. The two-line TGMS line Y58S was used as control. Y58S is a leading elite indica PTGMS line bred by Hunan Hybrid Rice Research Center and serves as the dominant female parent for two-line hybrid rice. All plants were grown in a standard greenhouse and in the paddy field at Fuzhou experimental station (26.08° N, 119.28° E), Fujian Province, China.

### 4.2. Vector Construction and Genetic Transformation

The full-length sequence of rice *MS1* (*Os09g0449000*) was retrieved from the Rice Genome Annotation Project (RGAP, http://rice.plantbiology.msu.edu/accessed on 6 May 2023). The sgRNA target sequence 5′-AAGCTGCTCAGCCTCGGCGA-3′ located in the exon region of *MS1* was selected for editing. An adenine base editor (ABE) vector targeting *MS1* (ABE-*MS1*) was constructed using the ABE8e-SpRY backbone. The homozygous mutant line GH89-*tms5* was generated by our research group in our previous study. *Agrobacterium tumefaciens* strain EHA105 harboring the respective vectors was used to transform mature embryo calli of GH89 and GH89-*tms5* following a standard protocol [14]. Potential off-target loci across the whole genome were predicted by bioinformatics tools, and specific primers were designed for PCR amplification and Sanger sequencing verification. The results demonstrated that no off-target mutations were detected at the *MS1* target site (Table S2).

### 4.3. Molecular Identification of Mutants

Genomic DNA was extracted from young leaves of T_0_ and T_1_ transgenic plants using the CTAB method. Transgenic positive plants were initially screened by PCR amplification of the *hygromycin phosphotransferase* gene (*HPT*). The target regions of *tms5* and *MS1* were amplified using gene-specific primers and subjected to Sanger sequencing to identify mutations. For the T_1_ generation, transgene-free mutants were identified based on the absence of the *HPT* gene and homozygosity at the target loci. Homozygous *MS1* single mutants and *tms5* + *MS1* double mutants were selected for subsequent phenotypic analysis.

### 4.4. Fertility Identification Assays

From June to mid-September 2025, the tms5, *MS1* single mutants and *tms5* + *MS1* double mutant in the GH89 background, together with the control line Y58S, were sown in seven batches at 20 day intervals. 100 individual plants were planted for each material. During the heading stage, 2 spikelets from the upper, middle and lower parts of panicles that were about to bloom were collected for pollen slide preparation. Spikelets were collected every 3 days. Pollen fertility was assessed by staining with 1% iodine-potassium iodide (I_2_-KI) solution and calculating the pollen fertility rate under a light microscope. For self-pollinated seed setting rate analysis, panicles at the same developmental stage were bagged before flowering, and the number of filled grains was counted at maturity. Plants were transferred to an artificial climate chamber (Conviron, Winnipeg, MB, Canada) when the main panicle reached young panicle differentiation stage VI. A constant average daily temperature of 23.5 °C was applied [15]. Plants were removed and transplanted back to the greenhouse after 5, 10, and 15 days of treatment. Pollen fertility rate and self-pollinated seed setting rate were measured as described in the Fuzhou experimental station (26.08° N, 119.28° E). For each plant, the highest values of pollen dark staining rate and bagged self-pollinated seed setting rate recorded across different observation periods were used as the fertility identification results for the corresponding treatment. In addition, 10 panicles of each plant were bagged before flowering, and the self-pollinated seed setting rate was investigated 25 days later. Meteorological data including daily average temperature were recorded simultaneously (Table S1).

### 4.5. Agronomic Trait Investigation and Statistical Analysis

At maturity, 8–10 representative plants of each genotype were selected to investigate major agronomic traits, including plant height, panicle length, effective panicles per plant and total grains per panicle. Experimental data were analyzed using the data processing system. Significant differences among groups were determined by one-way ANOVA followed by Tukey’s multiple comparison test. Data are presented as mean ± standard deviation (SD).

## 5. Conclusions

In this study, we successfully generated transgene-free *tms5* single, *MS1* single, and *tms5* + *MS1* double mutants in the *indica* rice variety GH89 using a combination of CRISPR/Cas9 and adenine base editing technologies. The dual mutant, GH89*-tms5 + MS1*, exhibited exceptional stability of male sterility, it maintained complete sterility for 84 consecutive days under natural field environments, and tolerated 10 days of continuous low-temperature stress (23.5 °C) in controlled experiments without fertility restoration. This superior performance is attributed to the non-overlapping fertility-sensitive periods of the two genes, which form a robust dual regulatory barrier against low-temperature-induced fertility restoration. Overall, this study provides a novel germplasm resource for two-line hybrid rice breeding and a new molecular strategy to ensure seed production safety under climate change.

## Figures and Tables

**Table 1:** Statistics on the types of *MS1* mutations in the T_0_ generation.

Transformation Material	No. of T_0_ Plants	Wild-Type Plants	No. of T → C Heterozygous Plants	No. of T → C Homozygous Plants
GH89	14	0	11	3
GH89-*tms5*	17	2	10	5

**Table 2:** The pollen viability and seed-setting rate of mutants under artificial climate chamber treatment. Five to ten panicles were randomly selected from each line for fertility detection in each treatment group. Values represent mean ± standard deviation. Different lowercase letters behind data indicate significant differences at the *p* < 0.05 level based on one-way analysis of variance (ANOVA).

Plants	Pollen Viability Rate (%)			Self-Pollinated Seed Setting Rates (%)		
	5 d	10 d	15 d	5 d	10 d	15 d
GH89-*tms5*-1	0.9 ± 0.5 b	15.2 ± 1.8 b	55.8 ± 3.1 b	0 b	3.8 ± 2.6 c	26.9 ± 12.8 b
GH89-*tms5*-2	1.1 ± 0.8 b	16.2 ± 3.6 b	57.6 ± 4.4 b	0 b	2.7 ± 1.5 c	27.7 ± 15.1 b
GH89-*tms5*-3	0.8 ± 0.6 b	15.5 ± 2.8 b	58.9 ± 4.2 b	0 b	3.0 ± 1.6 c	28.1 ± 13.2 b
GH89-*MS1*-1	45.3 ± 13.8 a	76.4 ± 17.0 a	89.6 ± 25.1 a	26.2 ± 10.5 a	41.8 ± 12.3 a	70.4 ± 13.1 a
GH89-*MS1*-3	41.8 ± 16.6 a	76.9 ± 18.6 a	87.3 ± 21.2 a	23.6 ± 7.8 a	45.5 ± 15.8 a	69.9 ± 11.4 a
GH89-*MS1*-5	43.7 ± 12.4 a	73.2 ± 15.3 a	91.2 ± 24.2 a	28.5 ± 15.3 a	45.2 ± 18.6 a	72.2 ± 15.2 a
GH89-*tms5* + *MS1*-2	0 c	0 c	10.2 ± 3.6 c	0 b	0 b	2.3 ± 1.9 c
GH89-*tms5* + *MS1*-12	0 c	0 c	11.8 ± 5.6 c	0 b	0 b	2.5 ± 1.7 c
GH89-*tms5* + *MS1*-13	0 c	0 c	10.1 ± 2.5 c	0 b	0 b	2.1 ± 1.5 c

**Table 3:** Pollen viability and self-pollination seed-setting rates of mutant lines and control Y58S under natural field conditions.

Heading Time	Pollen Viability Rate (%)				Self-Pollination Seed-Setting (%)			
	GH89-*tms5*	GH89-*MS1*	GH89-*tms5+MS1*	Y58S	GH89-*tms5*	GH89-*MS1*	GH89-*tms5+MS1*	Y58S
6 June 2025	79.5 ± 23.6 b	88.2 ± 23.9 a	38.2 ± 15.6 c	78.2 ± 15.9 b	39.6 ± 20.8 b	55.8 ± 22.4 a	15.4 ± 5.9 c	33.8 ± 13.9 b
9 June 2025	85.3 ± 26.5 a	72.4 ± 25.7 b	27.2 ± 12.8 c	82.5 ± 23.8 a	51.8 ± 28.9 a	51.2 ± 28.3 a	10.6 ± 8.8 b	48.2 ± 19.0 a
12 June 2025	70.6 ± 15.1 a	73.9 ± 16.8 a	30.1 ± 17.7 b	69.2 ± 13.6 a	32.3 ± 21.7 b	35.6 ± 18.7 b	12.3 ± 7.7 c	42.3 ± 25.6 a
15 June 2025	63.9 ± 27.6 a	26.1 ± 18.3 b	3.2 ± 2.6 c	60.7 ± 12.1 a	35.9 ± 12.3 a	8.8 ± 3.1 b	0 c	33.7 ± 18.8 a
18 June 2025	66.4 ± 22.9 a	28.1 ± 10.5 b	4.3 ± 2.5 c	59.8 ± 13.6 a	23.9 ± 5.4 a	7.2 ± 4.7 b	1.5 ± 0.7 c	21.9 ± 10.6 a
21 June 2025	23.5 ± 10.7 a	10.1 ± 4.5 b	1.3 ± 3.6 c	20.9 ± 3.2 a	4.8 ± 3.6 a	3.8 ± 2.6 a	0 b	3.1 ± 1.9 a
24 June 2025	8.7 ± 3.2 a	7.1 ± 2.1 a	0 b	8.5 ± 1.7 a	1.6 ± 1.2 a	1.4 ± 2.1 a	0 b	1.8 ± 1.1 a
27 June 2025	0 b	18.5 ± 10.4 a	0 b	0 b	0 b	5.4 ± 3.2 a	0 b	0 b
30 June 2025	0 a	0 a	0 a	0 a	0 a	0 a	0 a	0 a
3 July 2025	0 b	8.2 ± 3.5 a	0 b	0 b	0 b	2.1 ± 1.5 a	0 b	0 b
6 July 2025	0 b	7.1 ± 2.3 a	0 b	0 b	0 b	1.2 ± 0.6 a	0 b	0 b
9 July 2025	0 b	28.1 ± 5.9 a	0 b	0 b	0 b	8.7 ± 4.2 a	0 b	0 b
12 July 2025	0 b	4.1 ± 3.3 a	0 b	0 b	0 b	0.8 ± 0.5 a	0 b	0 b
15 July 2025	0 b	36.2 ± 12.6 a	0 b	0 b	0 b	12.4 ± 8.5 a	0 b	0 b
16 July 2025	0 b	56.3 ± 13.9 a	0 b	0 b	0 b	17.8 ± 9.1 a	0 b	0 b
18 July 2025	0 b	66.3 ± 28.7 a	0 b	0 b	0 b	25.9 ± 11.2 a	0 b	0 b
21 July 2025	0 a	0 a	0 a	0 a	0 a	0 a	0 a	0 a
24 July 2025	9.8 ± 6.4 a	0 b	0 b	8.5 ± 5.2 a	1.1 ± 0.5 a	0 b	0 b	1.8 ± 0.9 a
27 July 2025	6.9 ± 3.5 a	0 b	0 b	6.6 ± 3.3 a	0.5 ± 0.2 a	0 b	0 b	0.6 ± 0.3 a
30 July 2025	0 b	16.8 ± 8.4 a	0 b	0 b	0 b	5.2 ± 2.1 a	0 b	0 b
2 August 2025	0 a	0 a	0 a	0 a	0 a	0 a	0 a	0 a
5 August 2025	0 a	0 a	0 a	0 a	0 a	0 a	0 a	0 a
8 August 2025	0 a	0 a	0 a	0 a	0 a	0 a	0 a	0 a
11 August 2025	0 a	0 a	0 a	0 a	0 a	0 a	0 a	0 a
14 August 2025	0 a	0 a	0 a	0 a	0 a	0 a	0 a	0 a
17 August 2025	0 a	0 a	0 a	0 a	0 a	0 a	0 a	0 a
20 August 2025	0 a	0 a	0 a	0 a	0 a	0 a	0 a	0 a
23 August 2025	0 b	18.7 ± 5.2 a	0 b	0 b	0 b	3.5 ± 2.8 a	0 b	0 b
26 August 2025	0 b	24.9 ± 15.3 a	0 b	0 b	0 b	9.5 ± 3.1 a	0 b	0 b
29 August 2025	0 b	36.5 ± 18.9 a	0 b	0 b	0 b	10.8 ± 2.2 a	0 b	0 b
1 September 2025	0 b	42.6 ± 25.8 a	0 b	0 b	0 b	17.8 ± 10.6 a	0 b	0 b
4 September 2025	0 b	33.5 ± 15.3 a	0 b	0 b	0 b	10.3 ± 6.5 a	0 b	0 b
7 September 2025	0 b	41.7 ± 15.6 a	0 b	0 b	0 b	15.2 ± 8.1 a	0 b	0 b
10 September 2025	0 b	59.8 ± 25.4 a	0 b	0 b	0 b	35.4 ± 12.3 a	0 b	0 b
13 September 2025	0 b	38.9 ± 15.9 a	0 b	0 b	0 b	15.9 ± 9.1 a	0 b	0 b
16 September 2025	0 b	46.7 ± 5.43 a	0 b	0 b	0 b	25.2 ± 11.6 a	0 b	0 b

Pollen viability rate (%) and self-pollination seed-setting rate (%) were continuously monitored from June to September 2025. Each value represents the mean ± standard deviation (SD) of multiple independent plant replicates. The letters a, b, and c in the table indicate the results of multiple comparisons: identical letters mean no significant differences between groups, while different letters indicate significant differences.

## Data Availability

Data is provided within the manuscript or Supplementary Information Files.

## Supplementary Materials

The following supporting information can be downloaded at: https://www.mdpi.com/article/10.3390/plants15121906/s1. Table S1. Meteorological data at the Fuzhou experimental station during the field observation period, 2025-0515-0930. Table S2:Evaluation of potential off-target sites. Table S3. Primers used in this article.
